# Sequential combination therapy with flavopiridol and autocatalytic caspase-3 driven by amplified hTERT promoter synergistically suppresses human ovarian carcinoma growth *in vitro* and in mice

**DOI:** 10.1186/s13048-014-0121-3

**Published:** 2014-12-21

**Authors:** Yue Song, Xing Xin, Xingyue Zhai, Zhijun Xia, Keng Shen

**Affiliations:** Department of Obstetrics and Gynecology, ShengJing Hospital, China Medical University, No. 36, Sanhao street, Heping District, Shen yang 110004 China; Department of Obstetrics and Gynecology, Peking Union Medical College Hospital, Peking Union Medical College, Chinese Academy of Medical Sciences, No. 1, Shuai fuyuan Hutong, Dongcheng District, Beijing, China

**Keywords:** Amplified hTERT promoter, Autocatalysis, Caspase-3, Flavopiridol, Human ovarian carcinoma

## Abstract

**Background:**

Induction of cell apoptosis and regulation of cell cycle are very attractive for treatments of tumors including ovarian carcinoma. Flavopiridol is a potent small molecular cyclin-dependent kinase(cdk) inhibitor, but its antitumor efficacy is not satisfied yet. Caspase-3 play a major role in the transduction of apoptotic signals and the execution of apoptosis in mammalian cells. We have successfully constructed the recombinant adenovirues AdHTVP2G5-rev-casp3 containing autocatalytic caspase-3 (rev-caspase-3) driven by amplified hTERT promoter system (TSTA-hTERTp). In this study, we applied it with flavopiridol to investigate their antitumor effect on ovarian cancer in vitro and in vivo.

**Methods:**

Cell viabilities were determined using Cell Counting Kit 8 and flow cytometry. RT-PCR and immunoblotting assays were used to detect cellular apoptotic activities. Tumor growth and survival of mice bearing tumors were studied.

**Results:**

Flavopiridol or AdHTVP2G5-rev-casp3 at low dosage alone was mildly cytotoxic *in vitro* with a viability rate of 86.5 ± 4.7% for 300 nM flavopiridol and 88.9 ± 5.4% for AdHTVP2G5-rev-casp3 (MOI 20). By contrast, significant synergism of their sequential combination was observed, and the treatment of AdHTVP2G5-rev-casp3 (MOI 20) infection for 72 h, followed by flavopiridol (300 nM) for 48 h, can result in the most synergistic cell death, with cell survival rate and apoptotic rate of 11.6% and 69.7%, respectively. The sequential combination showed synergistic tumor suppression rate of 77.8%, which was significantly higher than that of AdHTVP2G5-rev-casp3 (33.6%) or flavopiridol (40.1%) alone. The mean survival of mice treated with the combination was 286 ± 8 d, which was synergistically longer than that of mice treated with AdHTVP2G5-rev-casp3 (141 ± 14d), flavopiridol (134 ± 10 d) or controls (106 ± 11 d) (*P* < 0.01).

**Conclusions:**

The sequential combination of rev-caspase-3 and flavopiridol result in significant synergistic cell killing effects, significant tumor growth suppression and extended survival of mice bearing OVCAR3 cells. The combination should be further explored as a potential clinically useful regimen against ovarian cancer.

**Electronic supplementary material:**

The online version of this article (doi:10.1186/s13048-014-0121-3) contains supplementary material, which is available to authorized users.

## Background

Deregulated cyclin-dependent kinase (cdk) activity is a hallmark of human cancer. Ectopic expression of cdk inhibitors in tumor cell lines usually results in cell cycle arrest in G1 or G2 or both, and this has translated into therapeutic benefit in xenograft models with slowed tumor growth and improved host survival [1]. Flavopiridol is a small molecule potent inhibitor of cdks, which is structurally related to a compound derived from the plant *Dysoxylumm binectariferum.* It strongly inhibits cdk1, cdk2, cdk4, and cdk7 and exerts cytostatic or cytotoxic activities against various human cancer cell lines. It also inhibits various kinases such as protein kinase A and C and epidermal growth factor-receptor tyrosine kinase at micromolar concentrations. It also broadly suppressed the transcription of genes, including cyclin D1, and binds to DNA [1-4]. To date, the results from phase II clinical trials of flavopiridol as a single agent are unsatisfactory [5]. Combination therapy of flavopiridol with other drugs have been attempted to improve the efficacy against various tumor types [6,7].

Caspase-3 plays a major role in the transduction of the apoptotic signal and execution of apoptosis in mammalian cells. There has been interest in using the pro-apoptotic caspase-3 gene for therapy against cancer. Yamabe *et al*. showed that caspase-3 transgene therapy in combination with an additional death-inducing therapeutic agent could be effective against various tumor types [8]. Shinoura *et al*. also showed that caspase-3 gene therapy required a pro-apoptotic signal to induce effective tumor cell killing [9]. Our group constructed constitutively active recombinant caspase-3 precursors (rev-caspase-3) that were capable of auto-processing and inducing significant apoptosis *in vivo* independent of the upstream caspases [10]. We also generate an amplified hTERT promoter system for efficient transcriptional targeting of active caspase-3 by using a two-step transcription amplification (TSTA) system. Our data have demonstrated a potent, hTERT-restricted apoptosis which is induced by adenovirus-mediated active caspase-3 gene driven by hTERTp-TSTA system in human ovarian cancer cells.

We hypothesized that adenovirus mediated delivery of constitutively active recombinant caspase-3 precursors to tumor cells in combination with flavopiridol may exhibit enhanced cytotoxicities against tumor cells when compared with either agent used alone. In the current study, we investigated the effect of the combination of flavopiridol and recombinant caspase-3 on OVCAR3 cells *in vitro* and in mouse xenograft model. We observed a very potent induction of apoptosis by the sequential combination treatment of these agents both at low doses in the cancer cells.

## Materials and methods

### Cell culture and drug preparation

Human ovarian adenocarcinoma cell line OVCAR3(Nanjing Biotechnology Development Co., Ltd.) was maintained in RPMI1640 (Gibco, Grand Island, NY) supplemented with 10% heat-inactivated fetal bovine serum (FBS) at 37°C with 5% CO_2_ in air. Flavopiridol, which was kindly provided by Professor Geoffrey I. Shapiro at the US National Cancer Institute, was prepared in dimethyl sulfoxide (DMSO) at a working concentration of 25 mM and stored at - 20°C and was diluted in fresh medium before use.

### Adenoviral vectors

AdHTVP2G5-rev-casp3, a recombinant adenoviral vector expressing rev-caspase-3 driven by the hTERTp-TSTA system, was constructed by our laboratory. Details of the recombinant vector are available upon request.

### Cell viability assays

OVCAR3 cells were seeded at a density of 1.7 × 10^4^ cells per well on 96-well plates and after an overnight incubation were treated with flavopiridol and/or AdHTVP2G5-rev-casp3 at the indicated doses. Cell viability was assessed using the Dojindo Cell Counting Kit-8 (Dojindo Laboratories, Gaithersburg, MD) according to the supplier’s recommendations. Absorbance was read at 450 nm and cell viability was expressed as the percentage of viable cells relative to untreated cells. All experiments were performed in triplicate and at least three times independently.

### Cell-cycle analysis

Treated OVCAR3 cells were washed once with phosphate buffered saline (PBS), trypsinized, and washed again in PBS with 2% FBS and then fixed in ice-cold ethanol for at least 1 h at −20°C. Then the cells were stained with propidium iodide (30 μg/ml) and treated with RNase (0.6 mg/ml) in PBS plus 0.5% (v/v) Tween20 and 2% FBS. Stained cells were analyzed on a FACS Calibur flow cytometer (BD Bioscience) using the Cellquest software, and the ModFit program (Verity Software House Inc, Topsham, ME) was used to analyze the cell-cycle profiles.

### Real time PCR

Total cellular RNA was isolated using the Trizol Reagent (Invitrogen, Carlsbad, CA). First-strand cDNA synthesis was carried out using reverse transcriptase (Invitrogen) by incubation at 25°C for 10 min, 37°C for 60 min and 95°C for 5 min. The sequences of primers used for real-time PCR were as follows: cyclin D: 5’-GAGGTCTGCGAGGAACAGAAGT-3’ (sense) and 5’-TTGAGCTTGTTCACCAGGAGC-3’ (anti-sense); active caspase-3: 5’-CCATGCTGAAACAGTATGCCG-3’ (sense) and 5’-TTCCAGAGTCCATTGATTCGCT-3’ (anti-sense); GAPDH: 5’-ACCACAGTCCATGCCATCAC-3’ (sense) and 5’-TCCACCACCCTGTTGCTGTA-3 (anti-sense). Amplification was performed in a 25-μL reaction containing 2 μL sample cDNA, 0.4 × *Taq*Man Universal PCR Master Mix (Applied Biosystems, USA), 120 nM each primer, and 1 nM DNA probe. The PCR was run at 94°C for 2 min followed by 40 cycles of 94°C for 5 s, 62°C for 10s and 60°C for 30s. Amplification was performed according to the manufacturer’s specifications.

### Immunoblotting studies

Cell lysates were prepared as previously described. Immunoblotting procedure was carried out as depicted earlier [11]. The following antibody was used for immunoblotting: anti-PARP antibody and anti-caspase-3 antibody (Cell Signaling Technology, Danvers, MA).

### Xenograft studies

The study protocol was approved by the local institution review board at the authors’ affiliated institution. Female mice, 4 to 6 weeks old and weighing 16 to 20 g (Animal Research Institution of Chinese Academy of Medical Sciences, Beijing, China), were used for the experiment. All animals were cared for according to the Guidelines for the Care and Use of Laboratory Animals and the institutional guidelines of Chinese Academy of Medical Sciences. For the subcutaneous tumor model, 1 × 10^7^ OVCAR3 cells in 0.2 ml PBS were inoculated subcutaneously into the dorsal flank of nude mice. When the tumor reached a volume of ~150 mm^3^, these mice received intratumoral injections of PBS, AdHTVP2G5-rev-casp3 (7 × 10^8^ tissue culture infectious dose 50 (TCID_50_)/tumor), flavopiridol (10 mg/kg) and the sequential combination treatment in which flavopiridol (5 mg/kg) was injected 72 h after treatment with AdHTVP2G5-rev-casp3 (7 × 10^8^ TCID_50_/tumor). Three intratumoral injections were given every 10 d and 5 mice from each group were followed up once per three days to measure tumor size by calipers. Tumor volumes were calculated using the formula *a* × *b*^2^ × 0.5, where *a* and *b* represent the larger and smaller diameters, respectively. Mice were sacrificed according to the institutional guidelines when the tumor reached 2000 mm^3^ in volume.

For the peritoneal tumor model, 1 × 10^7^ OVCAR3 cells in 0.5 ml PBS were injected intraperitoneally into the nude mice. Subsequently, mice were weighed once every two days. Twenty-one days after inoculation, the mice received intra peritoneal injections of PBS, AdHTVP2G5-rev-casp3 (7 × 10^8^ TCID_50_/tumor), flavopiridol (10 mg/kg) and the sequential combination treatment in which flavopiridol (5 mg/kg) was injected 72 h after treatment with AdHTVP2G5-rev-casp3 (7 × 10^8^ TCID_50_/tumor). Three intraperitoneal injections were given every 10 days. Survival was defined as the study endpoint. Histopathological changes in the liver, spleen, intestine, lung, kidney, ovary, pancreas and heart were examined and serum contents of alanine transaminase (ALT) and aspartate transaminase (AST) were performed as described previously [12]. Blood samples were collected via the tail vein on d 1 and 14 to monitor liver damage, and, specifically, the serum levels of AST and ALT in each group.

### Statistical analysis

Statistical differences among the treatment groups were assessed by ANOVA using SPSS11.5 software program. A value *P* < 0.05 was considered significant. Additionally, the survival data was summarized and plotted using the Kaplan-Meier method, and survival curves were compared using the log-rank test.

## Results

### Sequential combination treatment of flavopiridol and AdHTVP2G5-rev-casp3 suppresses the survival of OVCAR3 cells in vitro

**Figure 1:**
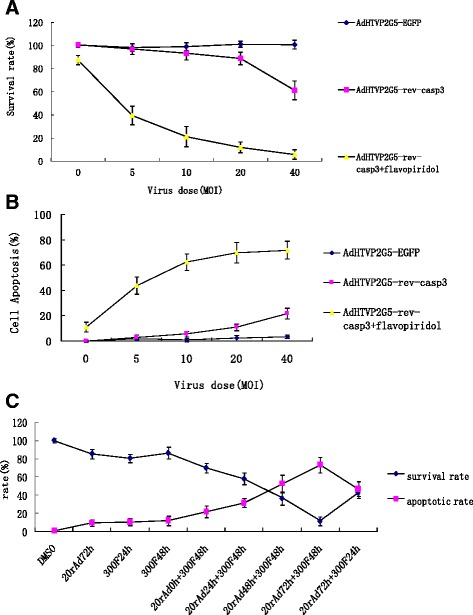
**Treatment with AdHTVP2G5-rev-casp3 sensitizes OVCAR3 cells to subsequent flavopiridol exposure. A** and **B**: Cell viability and apoptosis determined by CCK-8 assay and flow cytometry. OVCAR3 cells were infected by AdHTVP2G5-rev-casp3 alone at different MOIs as indicated, or 72 hs later, followed by flavopiridol (300 nM) for 48 hs. The results demonstrate the combination treatment can produce significant synergism of cell-killing effect using AdHTVP2G5-rev-casp3 at MOI of 5 ~ 40. The standard deviation for triplicate wells was less than 10%. **C**: Cells were infected with AdHTVP2G5-rev-casp3(MOI = 20) alone for 72 hs(rAd72 h), or 300 nM flavopiridol alone for 24 hs or 48 hs(300 F 24 h and 300 F 48 h, respectively). Alternatively, cells were infected with AdHTVP2G5-rev-casp3(MOI = 20) for 0 ~ 72 hs, followed by 300 nM flavopiridol for 48 hs (20 rAd 0 ~ 72 h + 300 F 48 h) or AdHTVP2G5-rev-casp3(MOI = 20) for 72 hs, followed by 300 nM flavopiridol for 24 hs(20 rAd 72 h + 300 F 24 h). Results demonstrate the sequential dependence of the AdHTVP2G5-rev-casp3/flavopiridol combination. Administration of the sequential 20 rAd 72 h + 300 F 48 h combination was more effective than other combinations.

We treated human ovarian adenocarcinoma cells OVCAR3 with 300 nM flavopiridol for 24 hs and then examined the viability of these cells using the CCK-8 method. We found that flavopiridol was only mildly cytotoxic with a viability rate of 86.5 ± 4.7%. Examination by flow cytometry of the sub-G1 population also found only a modest increase in the percentage of cells in the sub-G1 peak (10.3 ± 3.2% for 300 nM flavopiridol vs. 1.1 ± 2.9% for controls). Additionally, we infected OVCAR3 cells with AdHTVP2G5-rev-casp3 at an multiplicity of infection (MOI) of 5 to 40 for 72 h. Viability assays using the CCK-8 method showed that AdHTVP2G5-rev-casp3 caused a dose dependent suppression of the survival of OVCAR3 cells with 97.1 ± 4.8% of the cells viable at an MOI of 5 and 61.3 ± 8.1% of the cells viable at an MOI of 40 (Figure 1A). Flow cytometry further indicated a dose-dependent increase in the percentage of OVCAR3 cells undergoing apoptosis with an apoptotic rate of 3.1 ± 1.5% at an MOI of 5 and 21.8 ± 4.3% at an MOI of 40 (Figure 1B).

We next examined the effect of sequential combination treatment of AdHTVP2G5-rev-casp3 and flavopiridol on the proliferation of OVCAR3 cells. We treated OVCAR3 cells first with AdHTVP2G5-rev-casp3 at an MOI of 5 to 40 for 72 h followed by 300 nM for 48 h. Viability assays using the CCK-8 method showed that AdHTVP2G5-rev-casp3 at an MOI of 5 to 40 in combination with flavopiridol significantly suppressed the survival of OVCAR3 cells with a viability rate of 39.6 ± 7.9% at an MOI of 5 and 5.8 ± 4.1% at an MOI of 40 (Figure 1A), which was markedly lower than that of OVCAR3 cells treated with AdHTVP2G5-rev-casp3 and flavopiridol alone (*P* < 0.05). Flow cytometry additionally showed that the sub-G1 fraction increased to 43.4 ± 6.9% to 71.9 ± 7.0% in OVCAR3 cells treated with AdHTVP2G5-rev-casp3 at an MOI of 5 to 40 in combination with flavopiridol, which was markedly higher than that of OVCAR3 cells treated with AdHTVP2G5-rev-casp3 and flavopiridol alone (*P* < 0.05) (Figure 1B). These findings demonstrated that AdHTVP2G5-rev-casp3 and flavopiridol synergistically inhibited the survival of OVCAR3 cells by inducing significant apoptotic activities in these cells.

Moreover, the extent of cell death by the sequential combination was time dependent. We observed the cell survival rates when cells were infected by AdHTVP2G5-rev-casp3(MOI = 20), and then 0 ~ 72 h later, followed by flavopiridol (300 nM) for 24 ~ 48 hs. The results showed that the cell-killing synergism of them was the most potent when flavopiridol was added into cells at 72 h after the viral infection and lasted for 48 hs, in which the survival rate of OVCAR3 cells was 11.6 ± 4.6%,and the apoptosis rate was 69.7 ± 7.4% (Figure 1C).

### Sequential combination treatment of flavopiridol and AdHTVP2G5-rev-casp3 causes significantly enhanced apoptotic activities in OVCAR3 cells

**Figure 2:**
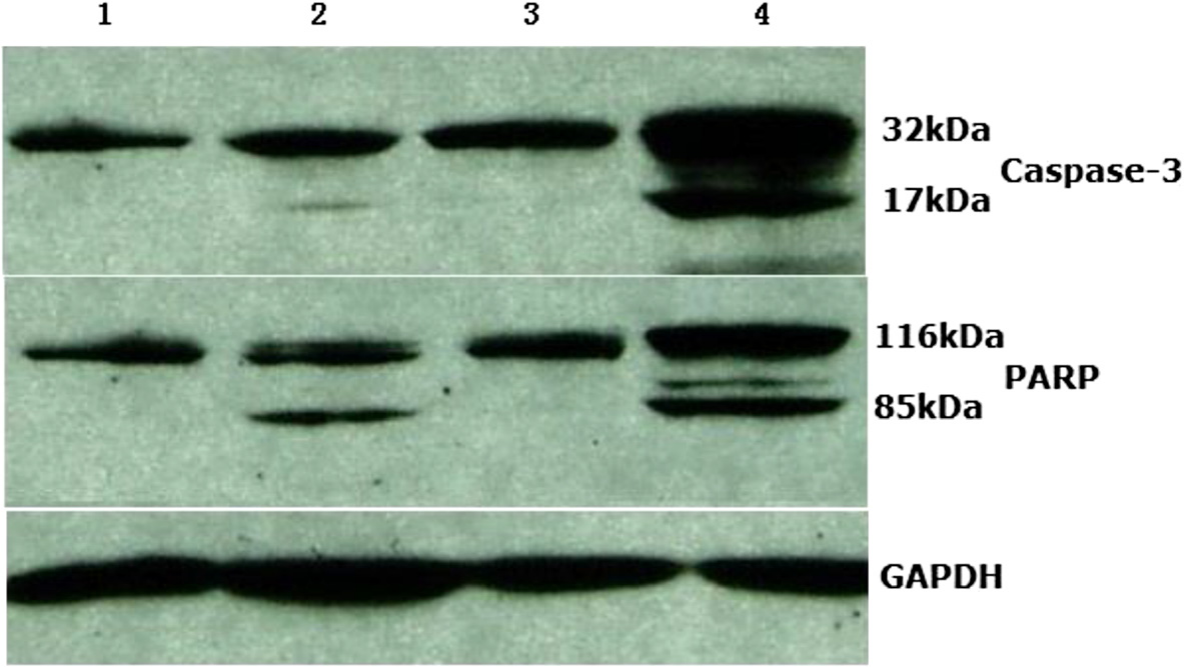
**Western blot analysis of apoptosis-related proteins.** The cells were plated in 100-mm plates and treated with AdHTVP2G5-rev-casp3/flavopiridol. Total protein extract of the cells was obtained, and Western blot analysis was performed as described in “Materials and methods.” Lane 1, untreated control; Lane 2, AdHTVP2G5-rev-casp3(MOI = 20) for 48 hs; Lane 3, 300 nM flavopiridol for 24 hs;Lane 4, AdHTVP2G5-rev-casp3(MOI = 20) for 48hs, followed by 300 nM flavopiridol. Western blot shown are representative of three independent experiments.

We further examined the levels of active caspase-3 in OVCAR3 cells treated with AdHTVP2G5-rev-casp3 (MOI = 20), 300 nM flavopiridol or their sequential combination by RT-PCR. We found that the mRNA transcript levels of active caspase-3 increased 55.82% in cells treated with AdHTVP2G5-rev-casp3 (3.95 ± 1.92) compared with controls (2.51 ± 1.56) (*P* < 0.05) while no apparent difference was found between the control cells and cells treated with flavopiridol (2.63 ± 1.15). The active caspase-3 mRNA levels were significantly higher in OVCAR3 cells subjected to the sequential combination treatment with AdHTVP2G5-rev-casp3 and flavopiridol (5.74 ± 2.43) than those of controls or cells treated with flavopiridol alone (*P* < 0.05 in both). Our immunoblotting analysis further revealed that the sequential combination treatment with AdHTVP2G5-rev-casp3 and flavopiridol caused significant increase in the levels of cleaved caspase-3 (p17) and cleaved PARP (p85) fragment (Figure 2). These findings further confirmed that AdHTVP2G5-rev-casp3 and flavopiridol caused significantly enhanced apoptotic activities in OVCAR3 cells.

### AdHTVP2G5-rev-casp3 of low dose causes accumulation of OVCAR3 cells in the S-phase and significantly enhances apoptosis induced by flavopiridol

**Figure 3:**
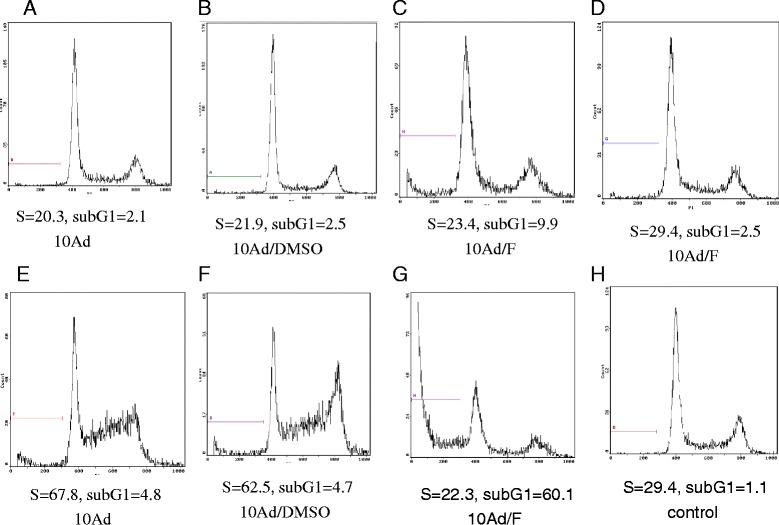
**Flow cytometric analysis in OVCAR3 cells.** **A-D**: Cells were treated with AdHTVP2G5-rev-casp3(MOI = 10), 36 h later, followed by DMSO or 300 nM flavopiridol for 48 hs. **E-H**, After treatment with AdHTVP2G5-rev-casp3(MOI = 10), 72 h later, cells were accumulated in S-phase. And then cells were subsequently treated with DMSO or 300 nM flavopiridol for 48 hs. Treatment with flavopiridol after recruitment to S-phase results in death of the majority of the cell population, inferred from the large percentage of cells with sub-G1 DNA content.

Flow cytometric analysis further showed that AdHTVP2G5-rev-casp3 (MOI = 10) caused a significant delay in S-phase progression with 130.6% increase in the percentage of OVCAR3 cells in the S-phase after the infection for 72 hs(67.8 ± 6.8% for AdHTVP2G5-rev-casp3 vs. 29.4 ± 5.2% for controls). Moreover, release into flavopiridol at 72 h after the viral infection by 48 h results in abrupt cell death of the majority of the population, compared with release into DMSO, in which cells continue to cycle (cell apoptosis rate of 60.1 ± 5.3% for the sequential combination of AdHTVP2G5-rev-casp3 and flavopiridol vs. 1.1 ± 2.9% for control). However, the cell cycle profile is not appreciably changed 36 h after cells were infected with AdHTVP2G5-rev-casp3(MOI = 10). Release into flavopiridol at this time point induces only a small amount of apoptosis compared with release into DMSO(cell apoptosis rate of 9.9 ± 4.6% vs. 2.5 ± 1.8%)( Table 1, Figure 3A-3H).

**Table 1:** **The apoptosis and cell cycle distributon of** ***OVCAR3*** **cells after the sequential combination treatment of AdHTVP2G5-rev-casp3(MOI = 10)for 36 hrs or 72 hrs followed by flavopiridol (300 nM) for 48 hrs**

	**Cell apoptosis rate (%)**	**Cell cycle distribution(%)**		
		**G1**	**S**	**G2/M**
Negative control	1.1 ± 2.9	51.7 ± 3.4	29.4 ± 5.2	18.9 ± 1.9
Flavopiridol(300nM) 24 hs	10.0 ± 3.2	63.8 ± 3.9	14.3 ± 1.5	21.9 ± 2.6
Ad. 36 hs	2.1 ± 0.3	52.6 ± 4.8	20.3 ± 2.7	27.1 ± 2.8
Ad.36 hs + DMSO 48 hs	2.5 ± 1.8	50.2 ± 3.8	21.9 ± 3.0	28.9 ± 3.1
Ad. 36 hs + Flavopiridol 48 hs	9.9 ± 4.6	48.5 ± 4.0	23.4 ± 3.1	28.1 ± 3.1
Ad.72 hs	4.8 ± 0.6	12.9 ± 2.1	67.8 ± 6.8*	19.3 ± 1.9
Ad.72 hs + DMSO 48 hs	5.2 ± 0.9	16.1 ± 2.6	62.5 ± 4.3	21.4 ± 2.2
Ad. 72 hs + Flavopiridol 48 hs	60.1 ± 5.3**	51.8 ± 3.8	22.3 ± 3.0	25.9 ± 3.1

*P < 0.01, **P < 0.01.

We examined the levels of cyclin D in OVCAR3 cells treated with AdHTVP2G5-rev-casp3, flavopiridol or their sequential combination by RT-PCR. We found no significant difference in cyclin D mRNA levels between control cells (0.69 ± 0.37) and those treated with AdHTVP2G5-rev-casp3 (0.72 ± 0.53). However, flavopiridol (300 nM) caused a marked reduction in the mRNA transcript levels of cyclin D (0.35 ± 0.31) compared with controls (*P* < 0.01). In addition, cyclin D mRNA levels were significantly lower in cells treated with AdHTVP2G5-rev-casp3 (MOI 20) in combination with flavopiridol (0.47 ± 0.39) than those of controls (*P* < 0.01).

### Sequential combination treatment of AdHTVP2G5-rev-casp3 with flavopiridol suppresses tumor growth and extends the survival of tumor xenograft-bearing mice

**Figure 4:**
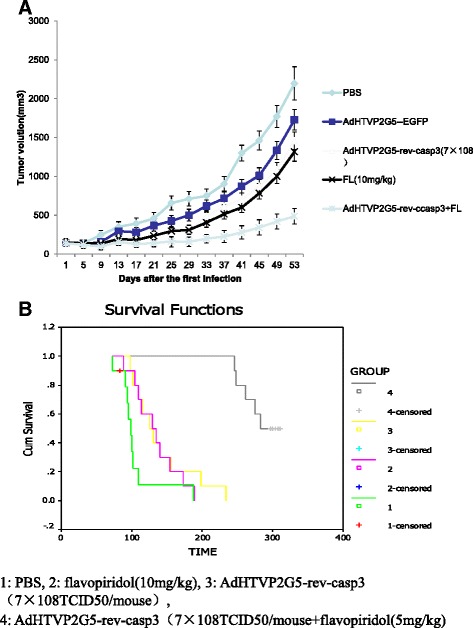
***In vivo*** **antitumor activity. A**: Subcutaneous tumors derived from OVCAR3 cells were treated as shown. Tumor volumes were monitored over time (days) after inoculation of tumor cells. Values represent the mean of 5 mice per group. **B**: 1:PBS, 2: flavopiridol (10 mg/kg), 3: AdHTVP2G5-rev-casp3 (7 × 10^8^ TCID50/mouse), 4: AdHTVP2G5-rev-casp3(7 × 10^8^ TCID50/mouse) + flavopiridol(5 mg/kg). Survival curves and mean survival durations for animals bearing abdominally spread OVCAR3 tumors. The animals received three treatments using PBS, flavopiridol, AdHTVP2G5-rev-casp3 or flavopiridol + AdHTVP2G5-rev-casp3 as described in the text. Survival was then monitored: the survival duration in animals receiving treatment using AdHTVP2G5-rev-casp3, flavopiridol or flavopiridol + AdHTVP2G5-rev-casp3 were significantly differently from those in the mice that received treatment using controls (*P* < 0.01). In addition, synergisms of AdHTVP2G5-rev-casp3 and flavopiridol were observed, and significantly prolonged survival can be achieved using combined AdHTVP2-G5-rev-casp3 and flavopiridol therapy without an increase in doses(*P* < 0.05).

To evaluate the *in vivo* efficacy of the sequential combination treatment of AdHTVP2G5-rev-casp3 with flavopiridol, we established mouse xenograft model by inoculating OVCAR3 cells subcutaneously in the nude mice and treated the mice with AdHTVP2G5-rev-casp3 and/or flavopiridol. Immunohistochemistry has confirmed the OVCAR3 xenograft. Fifty-three days after treatment, the tumor volume was 1458 ± 326, 1317 ± 295 and 487 ± 63 mm^3^ in mice bearing OVCAR3 xenograft treated with AdHTVP2G5-rev-casp3, flavopiridol and the sequential combination of AdHTVP2G5-rev-casp3 and flavopiridol, respectively. All treatments showed significant suppression of tumor growth at the end point of the study, with the tumor growth suppression rate of 33.6%, 40.1% and 77.8% *for* AdHTVP2G5-rev-casp3, flavopiridol and their combination, respectively *(P* < 0.05), as shown in Figure 4a. Moreover, we observed the synergism against xenograft growth between AdHTVP2G5-rev-casp3 and flavopiridol *in vivo.*

We next tested the effects of AdHTVP2G5-rev-casp3 and/or flavopiridol *in vivo* in an abdominally spread tumor model of OVCAR3 cells. Survival of mice in each group was recorded. Kaplan-Meier analysis indicated that AdHTVP2G5-rev-casp3, flavopiridol or sequential combination of AdHTVP2G5-rev-casp3 and flavopiridol significantly improved the survival of mice receiving intraperitoneal inoculation of OVCAR3 cells compared with control mice treated with PBS (*P* < 0.01) (Figure 4b). Furthermore, sequential combination of AdHTVP2G5-rev-casp3 and flavopiridol significantly prolonged survival of bearing tumor mice compared with that treated with AdHTVP2G5-rev-casp3 or flavopiridol alone. The mean survival of mice treated with sequential combination of AdHTVP2G5-rev-casp3 and flavopiridol was 286 ± 8 d with a median survival of 283 ± 7 d. This was significantly longer than the mean survival of mice treated with AdHTVP2G5-rev-casp3 (141 ± 14 d), flavopiridol (134 ± 10 d) or PBS (106 ± 11 d) (*P* < 0.01). Synergistic actions of AdHTVP2G5-rev-casp3 and flavopiridol were observed in extending the survival of the tumor-bearing mice with the mean and median survival of these mice almost doubled compared with mice treated with AdHTVP2G5-rev-casp3 or flavopiridol alone.

### Toxicity after intraperitoneal drug administration

**Figure 5:**
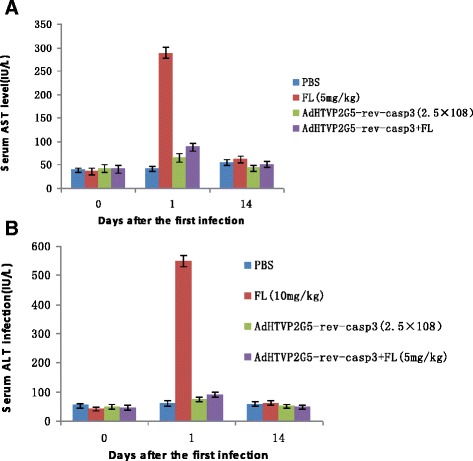
**Serum AST and ALT levels in abdominally spread tumor model mice after intraperitoneal drug administration.** **A** and **B**: Serum AST and ALT levels: Serum samples were collected before treatment started (day0), and one(day1) and 14 days(day14) after the last treatment.

We also examined the toxicity profiles of intraperitoneal administration of AdHTVP2G5-rev-casp3 and/or flavopiridol. We measured serum ALT and AST levels and found that they were only slightly elevated in mice receiving sequential combination of AdHTVP2G5-rev-casp3 and flavopiridol at low doses. The AST and ALT levels were significantly lower in mice receiving sequential combination of AdHTVP2G5-rev-casp3 and flavopiridol 1d after treatment than that in mice receiving 10 mg/kg flavopiridol (88.1 ± 7.8 vs. 289.3 ± 15.7, 91.5 ± 9.4 vs. 548.1 ± 42.9,respectively, *P* < 0.01 in both). On day 14, no significant difference in AST or ALT was seen among the groups (Figure 5a and 5b). We also examined the histopathological changes in the liver, spleen, intestine, lungs, kidneys, ovary and heart and we found no obvious lesions in mice receiving sequential combination of AdHTVP2G5-rev-casp3 and flavopiridol.

## Discussion

Cdk inhibitor flavopiridol has been demonstrated to exert potent antitumor activities in various preclinical tumor models [12,13]. It is possible that cdk inhibition by flavopiridol is primarily cytostatic in the majority of solid tumors not typically predisposed to apoptotic responses. In the study, we also found that flavopiridol was only moderately cytotoxic against human ovarian carcinoma cells *in vitro* but it demonstrated significantly improved survival of tumor-bearing mice compared with controls at 106 ± 11d post treatment. This survival advantage, however, disappeared at 134 ± 10 d post treatment, suggesting the modest activities of the agent. By contrast, the sequential combination treatment with AdHTVP2G5-rev-casp3 and flavopiridol not only showed synergistically enhanced cytotoxicities against human ovarian carcinoma cells *in vitro* but also significantly prolonged the survival of tumor-bearing mice with a median survival of 283 ± 7 d.

In our study, significant synergism with flavopiridol was observed when cells were treated with AdHTVP2G5-rev-casp3 at low doses. There is a very strong increase in cell death and the presence of a pronounced sub-G1 fraction, when the cells were infected by AdHTVP2G5-rev-casp3 at MOI of 5 ~ 20, followed by flavopiridol for 48 hs at 300 nM. The sub-G1 fraction increased to 43.4 ~ 71.9% and the cell survival rates decreased to 39.6% ~ 5.8%, accompanied with a significant decrease in S-phase content in the sequential combination treatment, compared with either flavopiridol or AdHTVP2G5-rev-casp3 treatment at drug concentrations above that alone produce almost no cell death. The cell-killing synergism of them was the most potent when the cells were infected by AdHTVP2G5-rev-casp3 at MOI of 20. However, the synergism became weak with the MOIs increased to more than 40, at which dose AdHTVP2G5-rev-casp3 treatment alone began to induce significant cell apoptosis with no recruitment of cells to S-phase. No significant synergism was observed when cells were treated at MOI of 70 ~ 100.

Moreover, the extent of cell death by the sequential combination was time dependent. We observed the cell survival rates when cells were infected by AdHTVP2G5-rev-casp3(MOI = 20), and then 0 ~ 72 h later, followed by flavopiridol (300 nM) for 24 ~ 48 hs. The results showed that the cell-killing synergism of them was the most potent when flavopiridol was added into cells at 72 h after the viral infection and lasted for 48 hs.

Utilization of hTERT promoter that is predominantly active in tumor cells would be an effective system to restrict rev-caspase-3 expression [14-16]. However, in most cancer cells, the hTERT promoter activity is more than 10-fold lower than that of the CMV promoter, and is too weak to achieve sufficient transgene expression. It has been shown that transgene expression from a tumor-specific promoter can be augmented by using TSTA system [17-19]. In the present study, we constructed the TSTA system using the hTERT promoter to drive a chimeric transcription factor consisting of the powerful herpes simplex virus VP16 transcriptional activation domain fused to the DNA-binding domain of the yeast protein GAL4, which then binds to the GAL4-binding sites upstream of a minimal promoter to activate rev-caspase-3 gene expression. Our studies showed that the TSTA system elevated the activity of the hTERTp up to a 200-fold in human ovarian cancer cell lines (data available upon request). Our *in vitro* and *in vivo* data about AdHTVP2G5-rev-casp3 demonstrated a strong, hTERT-restricted antitumor activity with significant reduction in liver toxicity.

It has been shown that cell lines can be sensitized to flavopiridol after recruitment to the S-phase. Apart from rev-caspase-3, treatment with non-cytotoxic concentrations of chemotherapeutic agents, including gemcitabine, cisplatin, and topoisomerase I and II inhibitors, are capable of retarding S-phase progression, suggesting that a common mechanism may exist for enhancing cytotoxicities against tumor cells by flavopiridol [20-22]. Our flow cytometric data showed here that AdHTVP2G5-rev-casp3 caused a significant increase in the percentage of OVCAR3 cells in the S-phase *in vitro*. We further showed that AdHTVP2G5-rev-casp3 sensitized OVCAR3 cells to flavopiridol with an apoptotic rate of 71.9% compared with no apparent apoptotic activities in OVCAR3 cells treated with either agent alone. We additionally found that the synergism between AdHTVP2G5-rev-casp3 and flavopiridol was sequence dependent and time dependent. It required that rev-caspase-3 precede flavopiridol by 72 hs. Exposure to flavopiridol preceding or concomitantly with rev-caspase-3 did not enhance cell death.

We have checked the levels of ALT and AST for their toxicity, and found that the acute toxicity by flavopiridol(10 mg/kg) alone was significantly increased after 1 days, much higher than that of the combination of flavopiridol (5 mg/kg) and AdHTVP2G5-rev-casp3 (2.5 × 10^8^). 10 mg/kg is the maximum tolerated dose(MTD) of flavopiridol *in vivo*, and this dose of flavopiridol can induce high toxicity levels in acute phase. But the combination of half of the MTD of flavopiridol (5 mg/kg) and low dose of AdHTVP2G5-rev-casp3 (7 × 10^8^ TCID50/mouse) induce much lower ALT and AST level than that in mice receiving 10 mg/kg flavopiridol alone (88.1 ± 7.8 vs. 289.3 ± 15.7, 91.5 ± 9.4 vs. 548.1 ± 42.9,respectively, *P* < 0.01 in both). This result showed that the combination of low dose flavopiridol and AdHTVP2G5-rev-casp3 can reduce the toxicity level, while maintain the synergism against xenograft growth *in vivo.*

In conclusion, we generated AdHTVP2G5-rev-casp3 and used it in sequential treatment with flavopiridol in OVCAR3 cells *in vitro* and in mouse xenograft model. We demonstrated that the sequential combination regimen exhibited synergistic cytotoxic activities against OVCAR3 cells *in vitro* and significantly inhibited tumor growth in mice and markedly extended the survival of tumor-bearing mice. Our findings indicate that the sequential combination regimen should be further explored as a potentially clinically useful treatment.

## Abbreviations

(ALT): Alanine transaminase
(AST): Aspartate transaminase
(cdk): cyclin-dependent kinase
(d): day
(FBS): Fetal bovine serum
(hs): hours
(MOI): Multiplicity of infection
(PBS): Phosphate buffered saline
(TCID): Tissue culture infectious dose
(TSTA): Two-step transcription amplification
(MTD): Maximum tolerated dose
